# Empfehlungen der Österreichischen Gesellschaft für Rheumatologie und Rehabilitation zu Ernährung und Lebensstil bei Gicht und Hyperurikämie – Update 2022

**DOI:** 10.1007/s00393-022-01286-2

**Published:** 2022-11-24

**Authors:** J. Sautner, G. Eichbauer-Sturm, J. Gruber, R. Lunzer, R. Puchner

**Affiliations:** 1 2. Med. Abteilung mit Schwerpunkt Rheumatologie, Niederösterreichisches Kompetenzzentrum für Rheumatologie, LK Korneuburg-Stockerau, Landstr.18, 2000 Stockerau, Österreich; 2 Rheumatologie und Nephrologie, Ordination für Innere Medizin, Linz, Österreich; 3 Univ. Klinik für Innere Medizin II, Med. Universität Innsbruck, Innsbruck, Österreich; 4 Rheumatologische Spezialambulanz, KH der Barmherzigen Brüder, Graz, Österreich; 5 Schwerpunktpraxis Rheumatologie und Gastroenterologie, Ordination für Innere Medizin, Wels, Österreich; 6 Medizinische Universität Wien, Wien, Österreich

**Keywords:** Adipositas, Patientenschulung, Internistische Komorbiditäten, Alkoholkonsum, Medizinische Beratung, Obesity, Patienteducation, Comorbidities, Alcohol consumption, Medical consultation

## Abstract

**Hintergrund:**

Gicht ist die häufigste entzündliche Gelenkerkrankung in der westlichen Welt und hat einen bewiesenen genetischen Hintergrund. Zusätzlich tragen Lebensstilfaktoren wie steigende Lebenserwartung und Wohlstand, ausreichende bzw. Überflussernährung und eine steigende Prävalenz von Adipositas in der Bevölkerung sowie z. B. Alkoholkonsum zur steigenden Inzidenz von Hyperurikämie und Gicht bei. Neben einer adäquaten medikamentösen Therapie ist fundierte medizinische Beratung zu Ernährung und Lebensstil ein essenzieller Teil des Managements von Gichtpatienten, die ein hohes Risiko für internistische Komorbiditäten haben.

**Ziel:**

Bereits 2015 hat der Arbeitskreis für Osteoarthritis und Kristallarthropathien in der Österreichischen Gesellschaft für Rheumatologie und Rehabilitation (ÖGR) Ernährungs- und Lebensstilempfehlungen für Patienten mit Gicht und Hyperurikämie publiziert. Seither wurde eine Vielzahl an Arbeiten zu diesem Thema publiziert, was ein Update notwendig machte.

**Methoden:**

Um die seit 2015 publizierte Literatur zu screenen, führten die Autoren zunächst eine hierarchische Literatursuche durch. Unter Berücksichtigung relevanter Zitate der ersten Publikation wurde die relevante Literatur ausgewählt, und die Empfehlungen aus 2015 wurden entweder beibehalten, umformuliert oder neu erstellt. Danach wurden der Evidenzgrad und der Grad der Zustimmung durch die Autoren für jede Empfehlung hinzugefügt.

**Ergebnisse:**

Auf dieser Basis wurden 10 Empfehlungen statt der bisherigen 9 erstellt. Wie in der Erstpublikation wurde eine grafische Darstellung mit Symbolen erstellt, um den Text nonverbal zu ergänzen.

**Schlussfolgerung:**

Die ÖGR-Empfehlungen zu Ernährung und Lebensstil für Patienten mit Gicht und Hyperurikämie wurden entsprechend dem aktuellen Stand der Literatur angepasst. Sie dienen als Informations- und Schulungsmaterial für Patienten und aktualisierte Information für Ärzte.

## Abstract (en)

**Background:**

Gout is the most frequent inflammatory joint disease in the western world and has a proven genetic background. Additionally, lifestyle factors, such as increasing life expectation and standard of living, sufficient or excess nutrition and a growing prevalence of obesity in the population as well as e.g. alcohol consumption, contribute to the rising incidence of hyperuricemia and gout. Apart from an adequate medication, medical consultation on nutrition and lifestyle is an essential part of the management of gout patients, who have a high risk of internal comorbidities.

**Objective:**

In 2015 the Austrian Society for Rheumatology and Rehabilitation (ÖGR) working group for osteoarthritis and crystal arthropathies published nutrition and lifestyle recommendations for patients with gout and hyperuricemia. Since then, a multitude of studies have been published addressing this topic, which necessitated an update.

**Methods:**

First, the authors performed a hierarchical literature search to screen for the literature published since 2015. Considering references given in the first publication, the relevant literature was selected and the recommendations from 2015 were either kept as published, reformulated or recreated. Finally, the evidence level and the level of agreement for each recommendation were added by the authors.

**Results:**

Based on this process, 10 recommendations were generated instead of the initial 9. As in the original publication, a graphical presentation with symbols was constructed to complement the written text.

**Conclusion:**

The ÖGR recommendations on nutrition and lifestyle for patients with gout and hyperuricemia were updated in accordance with the most recent relevant literature. These are supposed to serve as information and education material for patients and updated information for physicians.

## Einleitung

Mit einer Prävalenz von 2–3 % ist die Gicht die häufigste entzündliche Gelenkerkrankung in Europa und Nordamerika und zeigt eine steigende Inzidenz mit Alter und Wohlstand. Das macht die Gicht nicht nur zu einem allgemeinmedizinischen und rheumatologischen Gelenk- und schmerztherapeutischen Problem, sondern auch zu einem sozioökonomisch immer relevanter werdenden medizinischen Thema [1]. Gicht hat nicht nur das Potenzial der Chronifizierung und konsekutiven Gelenkzerstörung, sondern ist assoziiert mit kardiometabolischen renalen Problemen wie arterieller Hypertonie, Koronarer Herzkrankheit (KHK), Insult, Adipositas, Typ-2-Diabetes, Hyperlipidämie und chronischer Niereninsuffizienz [2, 3]. Eine Reihe von Empfehlungen diverser rheumatologischer Fachgesellschaften sowohl zur Diagnose als auch zur Therapie der Gicht liegt vor [4–8]. Gemeinsam mit der Medikation zu suffizientem Anfallsmanagement, Anfallsprophylaxe und Harnsäuresenkung sind Beratung zu optimierter Ernährung und Lebensstil Eckpfeiler in der Betreuung von Patienten mit Gicht und Hyperurikämie. Da die Krankheitslast der Gicht gut mit dem soziodemografischen Index korreliert, sind Ernährung und Lebensstil auch in diesem Kontext Themen, die bei einem optimalen und kompletten Gichtmanagement nicht fehlen dürfen [9].

Integraler Bestandteil aller aktuellen Leitlinien zum Management der Gicht sind – begleitend zur Medikation – auch Empfehlungen zu Ernährung und Lebensstilmodifikation. Trotz seit Jahren anhaltender Diskussionen ist die asymptomatische Hyperurikämie aus rheumatologischer Sicht nach wie vor keine Indikation für eine Harnsäure (HS) senkende Therapie, weil bis dato keine Evidenz dafür besteht, dass der Benefit der HS-Senkung in dieser Patientengruppe das mögliche Risiko der Medikation übertrifft. Dies unterstreicht das Potenzial und die Wichtigkeit von Ernährungs- und Lebensstilempfehlungen, um in einer ständig wachsenden Patientenpopulation mit Hyperurikämie (bedingt durch Alter, Übergewicht, Wohlstand u. a.) auch nicht medikamentös eine HS-Senkung herbeizuführen und zu unterstützen. Ein zusätzlicher wesentlicher Aspekt ist die potenzielle, positive Beeinflussung internistischer Komorbiditäten durch eine Ernährungsumstellung. Bei optimaler medikamentöser Behandlung und entsprechender Therapieadhärenz des Patienten ist Gicht prinzipiell gut zu therapieren und heilbar, in der Realität aber nach wie vor nicht suffizient behandelt [10]. Es ist mittlerweile gesichert, dass ohne entsprechende Änderung des Ernährungsverhaltens und ohne den Abbau von Übergewicht dies aber nicht möglich ist, was die nicht medikamentöse Komponente des Managements deutlich unterstreicht.

Empfehlungen, gerade wenn sie basale Bedürfnisse wie z. B. Ernährung betreffen, sollten generell lokalen bzw. nationalen Gegebenheiten angepasst werden, weil sie sonst naturgemäß oft unzureichend umsetzbar sind. Die regelmäßige Aktualisierung von evidenzbasierten Empfehlungen versteht sich von selbst und ist gerade in der Rheumatologie gelebte Praxis.

## Material und Methoden

Der Arbeitskreis für Osteoarthritis und Kristallarthropathien in der Österreichischen Gesellschaft für Rheumatologie und Rehabilitation (ÖGR) hat 2015 erstmalig Ernährungsempfehlungen für Patienten mit Gicht und Hyperurikämie erstellt und publiziert [11].

Aufgrund der Fülle an seit der Letztpublikation hinzugekommener Literatur ergab sich die Notwendigkeit für ein Update der Empfehlungen. Fünf Mitglieder des Arbeitskreises erklärten sich bereit, die hierarchische Literaturrecherche zu übernehmen und am Update mitzuarbeiten. Die Literaturrecherche wurde durch einen Medizinjournalisten unterstützt.

Im Zeitraum von März bis Juni 2021 wurden 160 Arbeiten mit Erscheinungsdatum bis 2021 (Suchzeitraum 01.01.2014–21.01.2021) über eine strukturierte PubMed-Suche identifiziert; 32 Publikationen wurden seit der letzten Literaturrecherche für die Empfehlungen aus 2014 berücksichtigt (damaliges Enddatum für Literaturrecherche 30.11.2013). Als Schlüsselwörter wurden Gicht/Hyperurikämie/Ernährung und Diät (gout/hyperuricemia/nutrition/diet) gewählt. Darunter stellte der Themenkomplex der DASH(Dietary Approaches to Stop Hypertension)-Diät den größten neu hinzugekommenen Anteil dar. Die DASH-Diät ist eine Diätform, die sich v. a. aus Obst, Gemüse, Milchprodukten mit niedrigem Fettgehalt sowie Vollkorn, Nüssen, Fisch und Geflügel zusammensetzt. Kaum enthalten sind darin Fett, rotes Fleisch und gezuckerte Getränke. Der bei dieser Diät niedrige Gehalt an Natrium, gesättigten Fettsäuren und Cholesterin sowie der andererseits hohe Gehalt an Kalium, Calcium, Magnesium und Fasern hat sich als effektiv in der Reduktion des Blutdrucks, aber auch von HS erwiesen [12, 13].

Es erfüllten 59 Arbeiten nach genauer Durchsicht und Grading gemäß dem Oxford-System die Kriterien für eine engere Auswahl; alle die Evidenz beeinflussenden Arbeiten wurden zitiert. Zusätzlich wurden relevante, rezent publizierte Arbeiten mit Fokus auf dem allgemeinen Einfluss von Lebensstilveränderungen, Diät und Körpergewicht nach Abschluss der Literaturrecherche einbezogen [14–16].

Alle Studien wurden von den Autoren gemäß deren Studiendesign mit Evidenzgrad (Oxford GRADE-System) kategorisiert (Tab. 1). Anhand dieser Literaturrecherche und Auswahl wurden die Empfehlungen von 2015 von den Autoren auf ihre Aktualität im Lichte der neu hinzugekommenen Literatur bewertet, nach Oxford GRADE graduiert und entweder unverändert belassen oder umformuliert bzw. neu erstellt [17]. Reviews bzw. Metaanalysen wurden bei Überlappungen in der Thematik für mehrere Empfehlungen herangezogen. Die Autoren beendeten ihre Arbeit schließlich mit 10 statt den bisherigen 9 Empfehlungen (Tab. 2). Nach Diskussion in der Gruppe erschien es den Autoren im Sinne des Duktus sinnvoller, eine Umgruppierung vorzunehmen und mit den allgemeinen Empfehlungen zu Gewichtsregulation und Diäten allgemein, gefolgt von Negativ- und Positivempfehlungen fortzufahren. Nach Ausformulierung wurde der Grad der Zustimmung mit jeder Empfehlung von 1–10 (1 = keine Zustimmung und 10 = volle Zustimmung) von allen Autoren erhoben und gemittelt.

**Table 1:** 

Grad der Empfehlung	Evidenzgrad	Studientyp
A	1a	Systematischer Review (homogener) randomisiert kontrollierter Studien
	1b	Einzelne randomisiert kontrollierte Studien (mit engen Konfidenzintervallen)
B	2a	Systematische Reviews (homogener) Kohortenstudien mit „exponierten“ und „nicht exponierten“ Probanden
	2b	Einzelne Kohortenstudie/„low-quality“ randomisiert kontrollierte Studien
	3a	Systematische Reviews (homogener) Fall-Kontroll-Studien
	3b	Einzelne Fall-Kontroll-Studien
C	4	Fallserien, „low-quality“ Kohorten- oder Fall-Kontroll-Studien
D	5	Expertenmeinungen, basierend auf nichtsystematischen Reviews oder mechanistischen Studien

**Table 2:** 

1	Gewichtszunahme und Übergewicht können den Harnsäurespiegel erhöhen und zu Gicht führen. Bei Übergewicht kann eine langsame Gewichtsabnahme (zumindest bei Männern) dazu beitragen, den Harnsäurespiegel zu senken und kann so vor Gicht schützen	
	*Evidenz: 2b (Grad B)*	*Grad der Zustimmung: 10*
2	Sowohl die Gicht als auch die Hyperurikämie sind mit kardiometabolischen und renalen Komorbiditäten assoziiert. Deswegen wird – begleitend zu Gewichtskontrolle und diätetischen Maßnahmen – regelmäßige körperliche Bewegung/Herz-Kreislauf-Training (150[–300] min/Woche mit moderater Intensität) empfohlen	
	*Evidenz: 2a (Grad B)*	*Grad der Zustimmung: 10*
3	Eine gesunde Ernährungsform wie die DASH(Dietary Approaches to Stop Hypertension)-Diät kann – in Kombination mit einer Gewichtsreduktion bei Übergewicht – Gicht, erhöhte Harnsäurespiegel und das kardiometabolische Risiko positiv beeinflussen	
	*Evidenz: 2b (Grad B)*	*Grad der Zustimmung: 9,8*
4	Rotes Fleisch, Innereien sowie Wurstprodukte können Harnsäurespiegel und Gichtrisiko erhöhen. Daher sollten v. a. rotes Fleisch und assoziierte Produkte nur selten (2-mal/Woche) und in geringen Mengen gegessen werden. Der Konsum von jeglichem – auch purinreichem – Gemüse wird ausdrücklich empfohlen	
	*Evidenz: 2b (Grad B)*	*Grad der Zustimmung: 10*
5	Meeresfrüchte (v. a. Krustentiere und Muscheln) können den Harnsäurespiegel und das Gichtrisiko erhöhen und sollten deswegen nur selten gegessen werden. Fisch als Bestandteil einer gesunden, Herz-Kreislauf-Erkrankungen vorbeugenden Diät wird regelmäßig (1- bis 2‑mal/Woche) empfohlen	
	*Evidenz: 3 (Grad B)*	*Grad der Zustimmung: 10*
6	Der Genuss von Alkohol erhöht das Gichtrisiko dosisabhängig. Vor allem Bier und Spirituosen sollten gemieden werden, während Rotwein das geringste Potenzial für ein erhöhtes Gichtrisiko darstellt	
	*Evidenz: 2a (Grad B)*	*Grad der Zustimmung: 10*
7	Gezuckerte Softdrinks, Fruchtsäfte und Lebensmittel mit hohem Fruktose- (Fruchtzucker‑)Gehalt können den Harnsäurespiegel erhöhen und sollten daher vermieden werden. Frisches Obst und fruchtzuckerfreie „Light-Getränke“ erhöhen das Gichtrisiko nicht	
	*Evidenz: 3 (Grad B)*	*Grad der Zustimmung: 9,8*
8	Regelmäßiger Genuss von (fettarmer) Milch/Milchprodukten kann die Harnsäure senken und ist allen Gichtpatienten zu empfehlen	
	*Evidenz: 1b (Grad A)*	*Grad der Zustimmung: 9,8*
9	Regelmäßiger Genuss von Kaffee kann helfen, den Harnsäurespiegel zu senken – auch in Ergänzung zu Diät und Medikamenten –, und ist daher zu befürworten	
	*Evidenz: 2b (Grad B)*	*Grad der Zustimmung: 9,6*
10	Kirschen (v. a. die Sorte Montmorency) sind in der Lage, den Harnsäurespiegel zu senken, indem sie die Ausscheidung über den Urin fördern. Allerdings ist noch unklar, in welcher Dosis die verschiedenen Produkte (Juice, Konzentrat, Extrakt) die beste Wirkung zeigen. Möglicherweise haben Sauerkirschen in Kombination mit Allopurinol einen komplementären Effekt	
	*Evidenz: 2b (Grad B)*	*Grad der Zustimmung: 9,0*

**Figure 1:**
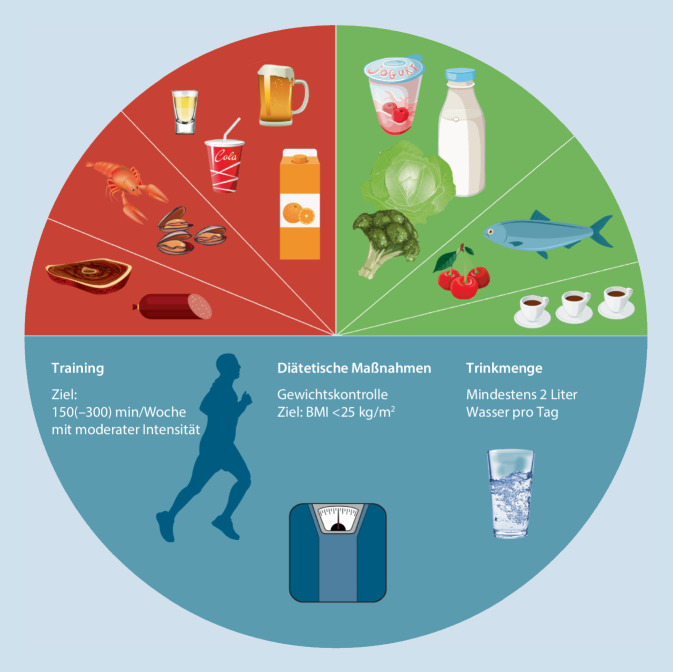

Die bereits 2015 erstellte grafische Darstellung der Empfehlungen mit Symbolen für eine effiziente rasche Übermittlung der Botschaft bzw. auch nonverbale Kommunikation mit Patienten bzw. deren Angehörigen wurde entsprechend adaptiert (Abb. 1).

## Ergebnisse

1. Gewichtszunahme und Übergewicht können den Harnsäurespiegel erhöhen und zu Gicht führen. Bei Übergewicht kann eine langsame Gewichtsabnahme (zumindest bei Männern) dazu beitragen, den Harnsäurespiegel zu senken, und kann so vor Gicht schützen.

**Evidenz 2b (Grad B) | Grad der Zustimmung: 10**

### Kommentar.

Diese Empfehlung wurde als aktuell und relevant erachtet und unverändert übernommen. Die Evidenz hat sich durch die hinzugekommene Literatur von 3 auf 2b gesteigert.

Beobachtungen an nordamerikanischen Patientenkohorten zeigen einen eindeutigen Zusammenhang von Übergewicht bzw. Adipositas mit Gicht sowohl bei Männern als auch bei Frauen. Die Prävalenz nimmt mit dem Grad der Adipositas zu. Aus großen prospektiven Beobachtungsstudien kann geschlossen werden, dass bei Männern mit Gicht *und* Übergewicht Gewichtsreduktion der Schlüsselfaktor zur Beherrschung der Gicht ist [18, 19]. In einer groß angelegten Studie bei über 12.000 Männern zeigte sich, dass eine Gewichtsabnahme dazu beitragen kann, bei Männern mit einem hohen kardiovaskulären Risiko eine Normalisierung der HS-Werte zu erreichen. Die Gewichtsabnahme bewirkte zwar eine geringere HS-Senkung als eine medikamentöse Therapie, hatte aber zusätzliche gesundheitliche Vorteile [20]. Ein BMI ≥ 25 kg/m^2^, Alkoholkonsum, das nicht Einhalten einer DASH-Diät und die Einnahme von Diuretika waren in dieser US-Studie mit 14.625 Erwachsenen dosisabhängig mit einer Hyperurikämie assoziiert. Allerdings war die entsprechende Varianz des Serum-HS-Spiegels, die durch diese Risikofaktoren erklärt wurde, sehr gering und konnte paradoxerweise ihre hohe Prävalenz zur Bewertung von Risikofaktoren in der Praxis nicht zeigen [21].

2. Sowohl die Gicht als auch die Hyperurikämie sind mit kardiometabolischen und renalen Komorbiditäten assoziiert. Deswegen wird – begleitend zu Gewichtskontrolle und diätetischen Maßnahmen – regelmäßige körperliche Bewegung/Herz-Kreislauf-Training (150[–300] min/Woche mit moderater Intensität) empfohlen.

**Evidenz 2a (Grad B) | Grad der Zustimmung: 10**

### Kommentar.

Diese Empfehlung wurde im Wording angepasst, aber sinngemäß aus 2015 übernommen. Hier hat sich der Grad der Evidenz von 3 auf 2a erhöht.

Die Gicht hat eine enge Beziehung zu Herz-Kreislauf- und Stoffwechselerkrankungen [2, 3]. Sie ist eng mit der Insulinresistenz verbunden und wird als Teil des metabolischen Syndroms angesehen. Doch Gicht bzw. Hyperurikämie sind nicht nur mit kardiometabolischen und renalen Komorbiditäten vergesellschaftet; Gichtpatienten haben eine erhöhte Mortalität. Es verwundert also nicht, dass die Bestimmung des Serum-HS-Spiegels im Rahmen der Abklärung einer arteriellen Hypertonie in den aktuellen Guidelines der European Society of Cardiology (ESC) empfohlen wird [22]. Ausmaß und Intensität der empfohlenen körperlichen Aktivität wurden in Anlehnung an die erwähnten ESC Guidelines gewählt, um eine Reduktion des kardiovaskulären Risikos, verbunden mit Gewichtsverlust und HS-Reduktion, zu forcieren. Der Trainingstyp wird dem Patienten überlassen, um die individuelle Motivation zu steigern. Ebenso wie Patienten mit kardiovaskulären Erkrankungen wird auch Patienten mit Gicht und Hyperurikämie regelmäßiges Herz-Kreislauf-Training empfohlen [20, 21]. Dies ist abseits der angestrebten HS-Senkung auch aus allgemeininternistischer Sicht stark zu befürworten.

3. Eine gesunde Ernährungsform wie die DASH(Dietary Approaches to Stop Hypertension)-Diät kann – in Kombination mit einer Gewichtsreduktion bei Übergewicht – Gicht, erhöhte Harnsäurespiegel und das kardiometabolische Risiko positiv beeinflussen.

**Evidenz 2b (Grad B) | Grad der Zustimmung: 9,8**

### Kommentar.

Diese Empfehlung ist aufgrund der Fülle an Literatur zur DASH-Diät neu hinzugekommen mit einem Evidenzgrad von 2b.

Die Wichtigkeit einer entsprechenden Diät bei Gicht und Hyperurikämie ist unbestritten [23]. Die differenzierte medikamentöse Therapie (Anfallsmanagement, Anfallsprophylaxe und HS-Senkung) ist in allen aktuellen Empfehlungen zur Behandlung der Gicht enthalten; der Stellenwert der Ernährungsberatung als wichtiger Bestandteil der Therapie wird zwar erwähnt, ist aber unterrepräsentiert.

Wesentliche Effekte der Gewichtsreduktion scheinen sich abseits der Reduktion der Adipositas durch die Reduktion der Insulinresistenz zu ergeben. Eine DASH-Diät scheint auch bei Patienten mit Gicht und Hyperurikämie eine HS-senkende Wirkung zu haben [24]. Eine wesentliche Studie widmet sich in diesem Zusammenhang dem unerlässlichen Aspekt der „patient education“. Sie konnte zeigen, dass eine umfassende Ernährungsberatung zu einer signifikanten Wissensverbesserung der Patienten unter einer harnsäuresenkenden Therapie bezüglich einer adäquaten Ernährung bei Gicht führt. Es konnte jedoch kein Unterschied der Serum-HS zur Kontrollgruppe, welche lediglich grundsätzliche Ratschläge bezüglich der Therapietreue und Nutzen einer Gewichtsabnahme erhalten hat, festgestellt werden [25]. Jedenfalls scheint eine Ernährungsberatung das Wohlbefinden der Patienten positiv zu beeinflussen.

Auch nicht primär auf Purinarmut fokussierte Ernährungsformen wie die Mittelmeerkost, Kalorienreduktion, Low-Carb und Low-Fat können wahrscheinlich durch eine Gewichtsreduktion und eine Senkung der Insulinresistenz eine Senkung der Serum-HS und zusätzlich der Blutfette (Cholesterin und Triglyceride) sowie der Insulinkonzentration bewirken und somit kardiovaskuläre Risikofaktoren positiv beeinflussen [26, 27]. Aus epidemiologischen Beobachtungen haben sich unter den bekannten Risikofaktoren die wichtigsten 4 für die Entwicklung einer Gicht herauskristallisiert: Übergewicht, Diätverhalten, Alkoholkonsum und Verwendung von Diuretika. Durch gesunde Ernährungsformen wie eine Mittelmeerdiät in Kombination mit einer Gewichtsabnahme bei Übergewichtigen und Adipösen können diese Risikofaktoren definitiv reduziert werden [28, 29]. Gemäß diesen Studien scheinen – nach dem Absetzen von Diuretika, was zu den häufigsten Ursachen für die Entwicklung einer sekundären Hyperurikämie und Gicht zählt – Übergewichtige am meisten von einer Diätintervention hinsichtlich HS-Senkung zu profitieren [29].

Eine DASH-Diät scheint einen identen Effekt zu haben, wobei dieser positive Einfluss hinsichtlich Senkung des HS-Spiegels besonders bei > 50-Jährigen, Frauen und bei körperlicher Inaktivität zum Tragen zu kommen scheint [30, 31]. In der Frage der zeitlichen Dimension des Effektes einer DASH-Diät scheint sich der HS-senkende Effekt bereits nach 1 Monat einzustellen und zumindest über 3 Monate anhaltend zu sein, umso ausgeprägter, je höher der Ausgangs-HS-Wert ist [24]. Studien zum Einfluss von DASH auf Gicht per se (z. B. Anfallshäufigkeit oder Tophi-Entwicklung) sind ausständig, aber die Senkung der Serum-HS als Surrogatparameter für die Gicht wird als definitives Argument für diese Diätform gewertet. Der initial für die Entwicklung dieser Diätform entscheidende Parameter, nämlich der blutdrucksenkende Effekt einer DASH-Diät, stellt naturgemäß ebenfalls einen positiven Faktor für Gichtpatienten dar. Man könnte in Anbetracht der Literatur eine DASH-Diät als attraktive präventive Diät für Männer mit einem Gichtrisiko bezeichnen [32]. In einer weiteren Studie bestätigte sich der HS-senkende Effekt einer DASH-Diät, insbesondere bei vorbestehender Hyperurikämie. Eine verstärkte Natriumzufuhr erwies sich hier ebenfalls als HS senkend – dieses Phänomen bedarf weiterer Studien zur Beurteilung [33].

Der Frage, ob das Alter eine Rolle für diätetische Interventionen spielt, wurde in der folgenden Studie nachgegangen: Auch in einem Kollektiv älterer Menschen (> 75 Jahre) ohne kardiovaskuläre Vorerkrankungen erwies sich eine mediterrane Diät als invers assoziiert mit dem Harnsäurespiegel, signifikant allerdings nur bei Männern (*p* = 0,02), den kardioprotektiven Effekt dieser Diät bestätigend [28].

4. Rotes Fleisch, Innereien sowie Wurstprodukte können Harnsäurespiegel und Gichtrisiko erhöhen. Daher sollten v. a. rotes Fleisch und assoziierte Produkte nur selten (2-mal/Woche) und in geringen Mengen gegessen werden. Der Konsum von jeglichem – auch purinreichem – Gemüse wird ausdrücklich empfohlen.

**Evidenz: 2b (Grad B) | Grad der Zustimmung: 10**

### Kommentar.

Diese Empfehlung aus 2015 wurde von den Autoren als aktuell und berechtigt eingestuft und nahezu unverändert übernommen. Neu hinzugekommene Literatur hat den Evidenzgrad von 3 auf 2b erhöht.

Entsprechend der aktuellen Literatur ist das Gichtrisiko in Bezug auf Ernährung im Wesentlichen vom Geschlecht unabhängig, sodass sich die Empfehlungen gleichermaßen auf beide Geschlechter beziehen.

Konsum von rotem Fleisch (Rind, Lamm, Schwein) bedeutet ein höheres Gichtrisiko: multivariates relatives Risiko (RR) = 1,41 (95 %-KI 1,07–1,86; *p* = 0,02) [34–37]. Eine wesentliche Erkenntnis ist die Unterscheidung von tierischen und pflanzlichen Purinen und die Unbedenklichkeit von pflanzlichen Purinen in jedem Gemüse, dessen Genuss jedenfalls forciert werden sollte [38].

5. Meeresfrüchte (v. a. Krustentiere und Muscheln) können den Harnsäurespiegel und das Gichtrisiko erhöhen und sollten deswegen nur selten gegessen werden. Fisch als Bestandteil einer gesunden, Herz-Kreislauf-Erkrankungen vorbeugenden Diät wird regelmäßig (1- bis 2‑mal/Woche) empfohlen.

**Evidenz: 3 (Grad B) | Grad der Zustimmung: 10**

### Kommentar.

Auch diese Empfehlung wurde als aktuell und relevant eingestuft und unverändert übernommen. Der Evidenzgrad blieb mit 3 gleich gegenüber 2015.

Für Meeresfrüchte und Krustentiere ergeben sich fast idente Daten wie für rotes Fleisch. (RR = 1,51; 95 %-KI, 1,17–1,95; *p* = 0,02). Insgesamt ergab sich ein erhöhtes Risiko für eine HS-Erhöhung bei Genuss von Meeresfrüchten, aber nicht von Fisch, ausgenommen fette Fische, wie z. B. Makrelen und Sardinen, sowie Fischhaut [35, 36]. Im Sinne einer gesunden Herz-Kreislauf-Diät wird in dieser Empfehlung dahingehend auch differenziert und der Genuss von Fisch empfohlen. Ein wesentlicher Aspekt in den Untersuchungen ist die Dosisabhängigkeit, was bedeutet, dass man neben seltenerem Verzehr auch zu kleineren Mengen raten sollte [37, 38].

6. Der Genuss von Alkohol erhöht das Gichtrisiko dosisabhängig. Vor allem Bier und Spirituosen sollten gemieden werden, während Rotwein das geringste Potenzial für ein erhöhtes Gichtrisiko birgt.

**Evidenz: 2a (Grad B) | Grad der Zustimmung: 10**

### Kommentar.

Diese Empfehlung wurde als aktuell und relevant eingestuft und wortgleich aus 2014 übernommen. Die hinzugekommene Literatur steigerte den Grad der Evidenz von 3 auf 2a.

Eine große prospektive Beobachtungsstudie in den USA zeigte mit einem Follow-up von 26 Jahren bei 44.654 Männern ohne Gicht in der Anamnese, dass das Gichtrisiko mit zunehmender Alkoholaufnahme zunahm (RR bei ≥ 30,0 g/Tag 2,10) [39]. Die Evidenz ist hier eindeutig.

Unter den verschiedenen Alkoholsorten hat sich die stärkste Assoziation mit erhöhtem Risiko für Gicht für Bier, gefolgt von Spirituosen, in der hinzugekommenen Literatur neuerlich gezeigt und somit erhärtet. In einer vielbeachteten Studie von Choi nahm Wein eine Sonderstellung ein und war nicht mit erhöhtem Risiko für Gicht assoziiert [40].

Gegenüber Alkoholabstinenten ist das multivariate RR für Männer, die 2 bis 3 Biere (1 Bier = 335 ml)/Woche trinken 1,27 (95 %-KI 1,00–1,62), und das RR steigt mit zunehmendem Bierkonsum (*p* < 0,0001). Bei einem Bierkonsum von ≥ 2 Bieren (entsprechend ≥ 670 ml/Tag) steigt das RR auf 2,51 (95 %-KI 1,77–3,55). Das multivariate RR für eine Zunahme um 1 Bier/Tag liegt bei 1,49 (95 %-KI 1,32–1,70). Eine japanische Arbeit untersuchte unterschiedliche Biersorten mittels Chromatographie, wobei sich lokale Biere (Privatbrauereien) bzw. auch alkoholfreies Bier als besonders purinreich herausstellten; Niedrigpurin- und Niedrigmalzbier bargen das geringste Gichtrisiko unter den Bieren [41]. Zusammengefasst zeigt sich bei Alkohol – ebenso wie beim Fleischkonsum – eine lineare Beziehung zwischen Frequenz der Zufuhr, Menge und Gichtrisiko. Verglichen mit Antialkoholikern liegt das altersangepasste RR bei einem Alkoholkonsum von 5–9,9 g/Tag bei 1,3 und steigt bei einem Konsum von 50 g/Tag auf 3,02 (*p* < 0,0001) [21, 39, 40].

Ein rezenter systematischer Review hat sich mit Rauchen und Alkoholkonsum bei Patienten mit rheumatischen und muskuloskeletalen Erkrankungen beschäftigt, unter anderem Gicht. Während Tabakkonsum keinen Einfluss auf Gichtanfälle oder HS-Spiegel zu haben scheint, zeigt Alkoholkonsum eine signifikante Assoziation zwischen Menge und Art der alkoholischen Getränke und dem Auftreten von Schüben [42]. Studien zum Einfluss von Ethnie und geografischem Wohnort, um kulturelle Unterschiede herauszuarbeiten, wären in diesem Zusammenhang interessant, fehlen aber bis dato.

7. Gezuckerte Softdrinks, Fruchtsäfte und Lebensmittel mit hohem Fruktose- (Fruchtzucker‑)Gehalt können den Harnsäurespiegel erhöhen und sollten daher vermieden werden. Frisches Obst und fruchtzuckerfreie „Light-Getränke“ erhöhen das Gichtrisiko nicht.

**Evidenz: 3 (Grad B) | Grad der Zustimmung: 9,8**

### Kommentar.

Diese Empfehlung aus 2015 wurde im Wording angepasst und präzisiert im Sinne der hinzugekommenen Literatur. Frisches Obst wurde – auch im Hinblick auf seinen Anteil an einer mediterranen Diät – explizit positiv bewertet, der Fokus von Getränken auf Lebensmittel erweitert, um auch Fruktose enthaltende Convenience-Produkte abzudecken. Der Grad der Evidenz ist mit 3 gleichgeblieben.

Aus Beobachtungsstudien an > 89.000 Probanden zeigt sich, dass Fruktose den HS-Spiegel signifikant erhöht und deswegen gemieden werden sollte [43–47]. In Anbetracht des mittlerweile fast ubiquitären Zusatzes von Fruktose abseits von Süßigkeiten haben v. a. Fertigprodukte („convenience food“) diesbezüglich ein Risikopotenzial [48, 49]. Speziell wird von Fruktose-reichen Fruchtsäften (v. a. Orangensaft) und süßem Obst (z. B. Orangen oder süße Äpfel) abgeraten. Im Gegensatz dazu sind Light- und Diätgetränke ohne Fruchtzucker unbedenklich hinsichtlich des Gichtrisikos. Auf andere wesentliche Aspekte, wie z. B. das Risiko für die Entstehung von Diabetes mellitus, wurde hier bewusst nicht eingegangen.

8. Regelmäßiger Genuss von (fettarmer) Milch/Milchprodukten kann die Harnsäure senken und ist allen Gichtpatienten zu empfehlen.

**Evidenz 1b (Grad A) | Grad der Zustimmung: 9,8**

### Kommentar.

Diese Empfehlung aus 2015 wurde als aktuell und relevant eingestuft und wortgleich übernommen. Die Evidenz ist mit 1b seit der Erstpublikation gleichgeblieben.

Für dieses Thema ergibt sich so wie 2015 die höchste Evidenz (1b) von allen für diese Empfehlungen ausgewählten Studien [36, 37].

In mehreren randomisierten Studien konnte der positive Effekt von Milch auf die Senkung des HS-Spiegels bzw. Gicht per se nachgewiesen werden [34, 35, 50–52].

Der Genuss von 250 ml Milch/Tag führte bei Männern zu einer Reduktion des Gichtrisikos um 50 %. Regelmäßiger Genuss fettarmer Milch und Joghurts führte zu einer 10 %-Senkung des HS-Spiegels. Ursächlich für den positiven Effekt ist der HS-senkende Effekt der Milchproteine Casein und Lactalbumin. Sojamilch führte hingegen interessanterweise zu einem 10 %-Anstieg des HS-Spiegels.

9. Regelmäßiger Genuss von Kaffee kann helfen, den Harnsäurespiegel zu senken – auch in Ergänzung zu Diät und Medikamenten –, und ist daher zu befürworten.

**Evidenz: 2b (Grad B) | Grad der Zustimmung: 9,6**

### Kommentar.

Diese Empfehlung wurde als aktuell und relevant eingestuft und bis auf leichte Änderungen im Wording unverändert beibehalten. Der Grad der Evidenz hat sich durch neu hinzugekommene Literatur von 3 auf 2b gesteigert.

Die Mechanismen, die der Wirkung von Kaffee auf den Serum-HS-Spiegel zugrunde liegen, sind letztendlich noch nicht eindeutig definiert. Mehrere mögliche Erklärungen werden diskutiert. Koffein (1,3,7-Trimethylxanthine) im Kaffee hemmt die Xanthinoxidase, erhöht die Nierendurchblutung und verbessert die Ausscheidung von Urat im Urin. Sowohl koffeinhaltiger als auch koffeinfreier Kaffee enthalten Chlorogensäure, wodurch die Insulinresistenz verbessert und dadurch der Serum-HS-Spiegel gesenkt werden kann [53–56].

Entsprechend der Literatur senkt Koffein das Gichtrisiko [36, 37].

Eine Korrelation zwischen Kaffeekonsum und dem Risiko einer Hyperurikämie wird unterschiedlich bewertet. Eine Metaanalyse aus 2016 kam zu dem Schluss, dass – bei limitierter Evidenz aufgrund weniger Studien – Kaffeekonsum möglicherweise mit einem niedrigeren Risiko für Gicht assoziiert ist, fordert aber gleichzeitig gut geplante weitere Studien [52]. Die Ergebnisse einer Metaanalyse aus 2018 ergaben keine Wechselbeziehung zwischen Kaffeekonsum und Serum-HS-Spiegel bei Männern, aber ein diesbezüglich erhöhtes Risiko für Frauen [36]. Auch wenn regelmäßiger Kaffeekonsum die Inzidenz von Gicht senkt und unterstützend wirken kann, ist Kaffee kein wirksames alleiniges Instrument zur Senkung des HS-Spiegels, sondern lediglich als supportiv zu bewerten.

10. Kirschen (v. a. die Sorte Montmorency) sind in der Lage, den Harnsäurespiegel zu senken, indem sie die Ausscheidung über den Urin fördern. Allerdings ist noch unklar, in welcher Dosis die verschiedenen Produkte (Juice, Konzentrat, Extrakt) die beste Wirkung zeigen. Möglicherweise haben Sauerkirschen in Kombination mit Allopurinol einen komplementären Effekt.

**Evidenz 2b (Grad B) | Grad der Zustimmung: 9,0**

### Kommentar.

Diese Empfehlung wurde neu formuliert bzw. ersetzt die Empfehlung zum Genuss von Vitamin C aus 2015, weil die Evidenz für Vitamin C in der Zwischenzeit als nicht mehr aktuell eingestuft werden muss.

Unter den pflanzlichen Nahrungsmitteln mit einem HS-senkenden Potenzial findet sich die meiste Literatur für Kirschen. Sauerkirschen, die sehr viel Anthocyane enthalten, werden viele gesundheitsfördernde Effekte zugeschrieben. Die existierenden Studien zur Messung ihres Potenzials, die HS-Ausscheidung zu fördern, sind widersprüchlich und Gegenstand von Diskussionen, was sich im niedrigsten Grad der Autorenzustimmung (9,0) unter den 10 Empfehlungen widerspiegelt [57–59]. Die aktuelle – wenn auch sehr dürftige – Studienlage unterstützt einen Zusammenhang zwischen dem Verzehr von Kirschen und einem dadurch geringeren Risiko von Gichtanfällen [60–63]. Deswegen wurden Kirschen auch in die Abbildung mit aufgenommen, was nicht als Empfehlung der Autoren für den Verzehr von Nahrungsergänzungsmitteln aus Sauerkirschen zu werten ist. Weitere umfassende Studien sind erforderlich, um die Wirksamkeit des Verzehrs von Kirschen bei der Behandlung von Patienten mit Gicht oder Hyperurikämie zu bewerten. Langzeiteffekte und der exakte Wirkmechanismus für die Reduktion von Gichtattacken sind von Interesse. Das Ergebnis einer laufenden Untersuchung von Lamb und Kollegen bleibt abzuwarten. Bei dieser Studie handelt es sich um die erste randomisierte, doppelblinde, placebokontrollierte Studie zur Untersuchung der Wirksamkeit von Sauerkirschsaft zur Senkung des Risikos eines erneuten Gichtanfalls [64]. Die Ergebnisse könnten entscheidend für die zukünftige Einordnung sein.

## Diskussion

Zur Wichtigkeit und zum Nutzen eines gesunden Lebensstils mit ausgewogener Ernährung, Gewichtskontrolle und körperlichem Training bei Menschen mit muskuloskeletalen Erkrankungen liegen belastbare Daten vor [14]. Der Einfluss von Training und Körpergewichtskontrolle im Hinblick auf eine Lebensstiländerung ist gut belegt, auch für Gichtpatienten [15]. Es besteht breiter Konsens, dass Lebensstiladaptierungen, auch wenn sie essenzieller Bestandteil des Managements von Patienten mit muskuloskeletalen Erkrankungen sind, das medikamentöse Management unterstützen, aber nicht ersetzen können. Lebensstilempfehlungen sollten – so wie die Medikation – auf den Patienten individuell zugeschnitten werden und Faktoren wie Alter, Geschlecht, Gesundheitszustand und Komorbiditäten berücksichtigen.

Ziel dieser Arbeit war eine evidenzbasierte Aktualisierung der Empfehlungen zur nichtmedikamentösen Senkung des Harnsäurespiegels. Diese immer noch zu wenig beachtete Therapiesäule stellt für Patienten mit Gicht, der immer eine Hyperurikämie zugrunde liegt, einen wesentlichen Bestandteil zur kausalen Intervention dar.

Wie bei den ersten Empfehlungen im Jahr 2015 entschieden sich die Autoren, gemeinsame Empfehlungen für Patienten und Ärzte zu erstellen [11]. Diese kombinierten Empfehlungen sind dazu gedacht, einerseits den Kollegen mit diesem Update den aktuellen Stand des Wissens inklusive Evidenzgrad und inkludierter Literatur zur Kenntnis zu bringen und gleichzeitig anschauliche und verständliche Formulierungen und Bilder als optimale Information für Patienten zu bieten. Die Farbwahl entspricht wiederum dem allgemein gebräuchlichen und international verständlichen Ampelsystem, sprich rot für zu meidende bzw. zu reduzierende Nahrungsmittel, grün für Erlaubtes und blau für allgemeine Ernährungs- und Lebensstilempfehlungen. Zur optimalen Veranschaulichung wurde die Gliederung wieder in einen schriftlichen Teil auf der Vorderseite und eine bildliche Darstellung mit Symbolen (Kreisform) auf der Rückseite gewählt – mit identischer Farbgebung zu den auch farblich markierten schriftlichen Empfehlungen. Die bildliche Darstellung ist auch für Patienten mit Sprachbarriere geeignet. Die Reihung der Empfehlungen wurde im Sinne des Duktus geändert: Die 3 allgemeinen Empfehlungen mit der Betonung der Wichtigkeit von Gewichtsreduktion bei Übergewicht und Halten von Normalgewicht bzw. der generellen Favorisierung einer gesunden Ernährungsform wurden an den Anfang gestellt. Darauf folgen nun die 4 Empfehlungen, die Nahrungs- und Genussmittel thematisieren, die zu meiden bzw. zu reduzieren sind. Den Abschluss stellen die 3 Empfehlungen mit zu forcierenden, weil den HS-Spiegel senkenden Nahrungsmitteln dar.

Der Grad der Evidenz hat sich bei 5 Empfehlungen durch die neu hinzugekommene Literatur im Vergleich zu 2015 gesteigert [11]. Für die einzelnen Empfehlungen wurden die Evidenzgrade mit 1‑mal 1b (Empfehlung 8, fettarme Milchprodukte), 7‑mal 2(a/b) und 2‑mal 3 (Empfehlung 5, Meeresfrüchte und 7, Fruktose) ermittelt. Die Auswahl der Arbeiten erfolgte durch die Mitglieder des Arbeitskreises. Den Empfehlungen zugrunde liegen maßgebliche Publikationen aus der verfügbaren Literatur, wobei alle prospektiv randomisierten Studien zu den betreffenden Themen sowie große epidemiologische Untersuchungen mit klinischer Relevanz inklusive vorliegender Metaanalysen und Reviews eingeschlossen worden sind. Die hierarchische Literatursuche wurde unter Anwendung der Oxford GRADE-Regeln durchgeführt, allerdings ohne Anwendung einer methodologischen Qualitätsbewertung für systematische Reviews (z. B. AMSTAR = Bewertungsinstrument, um systematische Reviews zu bewerten).

Der Fokus wurde – wie bei den ersten Empfehlungen aus 2015 – auf Praxisbezug gelegt, um den Bedürfnissen von Patienten und klinisch tätigen Kollegen zu entsprechen. Bei den Empfehlungen wurde bewusst nicht zwischen Gicht und Hyperurikämie differenziert, weil hier beide Patientengruppen angesprochen werden sollen – mit dem übergeordneten Ziel der HS-Senkung. Anstelle von 9 Empfehlungen im Jahr 2015 gibt es in der aktualisierten Fassung 10 Empfehlungen. Dazugekommen sind die Empfehlungen 3 (DASH-Diät) und 10 (Montmorency-Kirschen). Nicht mehr enthalten ist die Vitamin-C-Gabe für Gichtpatienten aufgrund unzureichender Daten [23]. Die wesentlichen neuen Aspekte hinsichtlich Gewichtsreduktion und DASH-Diät wurden berücksichtigt.

Wesentliche Ergebnisse aus Studien der letzten Jahre zeigen, dass eine kontrollierte Gewichtabnahme nicht nur sowohl den Blutdruck als auch das kardiovaskuläre Risiko senkt, sondern auch als ein wichtiger Faktor für eine diätetische HS-Senkung und eine Verminderung des Risikos für die Entwicklung einer Gichterkrankung angesehen werden kann [18–21]. Diät und Lebensstil allein können Gicht nicht heilen, was auch keinem Patienten suggeriert werden soll [16]. Die HS-senkende Therapie ist und bleibt der Eckpfeiler der Gichttherapie, aber das Ansprechen von Ernährung und Lebensstil komplettiert bei diesen Patienten – in Anlehnung an die erwähnten Empfehlungen der Fachgesellschaften – neben der medikamentösen Versorgung das Gichtmanagement [65]. Wichtig ist ferner die Betonung der Dosisabhängigkeit für alle erwähnten Nahrungs- und Genussmittel, weswegen eine wesentliche Botschaft die Vermeidung von Exzessen ist. Ein Ziel ist es, den Patienten zu vermitteln, dass den HS-Spiegel steigernde Nahrungs- und Genussmittel nicht für immer kategorisch verboten sind, sondern auch weiterhin – aber in kleineren Mengen bzw. seltener – genossen werden können. Das ist eine wesentliche Strategie, Compliance und Adhärenz zu erhöhen. Abschließend ist die Beibehaltung von 8 der ursprünglichen 9 Empfehlungen als Beweis für die solide Evidenzbasis bereits für die Erstpublikation 2015 zu werten. Die Fülle an seit der Letztpublikation hinzugekommener Evidenz zur DASH-Diät wurde in Form einer neuen Empfehlung hinzugefügt. Schlussendlich wurde die grafische Aufbereitung an die neuen Aspekte der Empfehlungen angepasst. Die Empfehlungen aus 2015 sind aufgrund der großen Nachfrage im niedergelassenen Bereich bereits in 10 Sprachen übersetzt worden (Englisch, Arabisch, Bulgarisch, Chinesisch, Farsi, Kroatisch, Serbisch, Rumänisch, Ungarisch und Türkisch). Eine Übersetzung der aktualisierten Empfehlungen kann für die Zukunft angedacht werden.
